# Uniqueness of Solutions in Thermopiezoelectricity of Nonsimple Materials

**DOI:** 10.3390/e24091229

**Published:** 2022-09-01

**Authors:** Francesca Passarella, Vincenzo Tibullo

**Affiliations:** Dipartimento di Matematica, Università di Salerno, 84084 Fisciano, Italy

**Keywords:** thermopiezoelectricity, nonsimple materials, Green and Laws, uniqueness

## Abstract

This article presents the theory of thermopiezoelectricity in which the second displacement gradient and the second electric potential gradient are included in the set of independent constitutive variables. This is achieved by using the entropy production inequality proposed by Green and Laws. At first, appropriate thermodynamic restrictions and constitutive equations are obtained, using the well-established Coleman and Noll procedure. Then, the balance equations and the constitutive equations of linear theory are derived, and the mixed initial-boundary value problem is set. For this problem a uniqueness result is established. Next, the basic equations for the isotropic case are derived. Finally, a set of inequalities is obtained for the constant constitutive coefficients of the isotropic case that, on the basis on the previous theorem, ensure the uniqueness of the solution of the mixed initial-boundary value problem.

## 1. Introduction

The classical theory of heat propagation is based on two equations: the energy balance equation and Fourier’s law for the heat flux. These equations lead to the classical heat equation which has the unrealistic characteristic that the speed of heat propagation is infinite. This classical theory was initially questioned by Cattaneo [1,2] and Vernotte [3], leading to the emergence of new fields of research: heat waves and second sound propagation can be found in the work of Straughan [4] and in extended irreversible thermodynamics (EIT) in the books of Jou et al. [5] and Müller and Ruggeri [6]. In [7], Gurtin and Pipkinconsidered a general theory of heat conduction with finite velocity, based on a law of heat flux with memory. For more information on theories of thermoelasticity that predict a finite velocity for the propagation of thermal signals, see the reviews of Chandrasekharaiah [8,9]. One of these theories is proposed by Green and Laws [10]. As presented by Ieşan [11], they make use of an entropy inequality in which a new constitutive function appears with the role of thermodynamic temperature (see, e.g., [12]). In addition to the finite velocity of heat waves, this theory also results in a symmetric tensor of thermal conductivity.

The origin of the theory of nonsimple elastic materials goes back to the works of Toupin [13,14], and Mindlin [15]. Toupin and Gazis [16] applied the general theory of grade 2 materials to the problem of surface deformation of a crystal. They showed that initial stress and *hyperstress* in a uniform crystal resulted in a deformation of a thin boundary layer near a free surface as had been observed in electron diffraction experiments. The theory of strain gradient of thermoelasticity is first presented in [17,18]. Elasticity gradient theory becomes important because it is adequate to study problems related to dimension effects and nanotechnology. In the micron and nano-scales regime, experimental evidence and observations have suggested that classical continuum theories are not sufficient for an accurate and detailed description of the corresponding deformation phenomena.

Furthermore, Kalpakides and Agiasofitou [19] have established a theory of electroelasticity including both the strain gradient and the electric field gradient. They report that taking into account the second spatial gradient of motion makes sense especially in crack problems, moreover taking into account the second gradient of electrical potential implies the presence of quadrupole polarization in the continuum model, of practical interest for surface effects problems The theory of nonsimple thermoelastic materials has been discussed in various articles (see for example [19,20,21,22,23,24,25,26,27]).

The question of the interaction of electromagnetic fields with elastic solids has been the subject of important investigations (see, e.g., [28,29,30,31,32,33] and the literature cited therein). Some crystals (such as quartz) subjected to stress, become electrically polarized (piezoelectric effect). Conversely, an external electromagnetic field produces deformation in a piezoelectric crystal. The theory of thermopiezoelectricity has been studied in various works (see, e.g., [34,35,36,37,38]).

To deepen the concept of thermodynamic entropy, the reader can refer to the review article [39]. In addition, for an application to piezoelectric phenomena see [40], where the authors study a piezoelectric energy harvester driven by random broadband vibrations and propose an analytical and numerical analysis of the linear model compatible with the experiments.

In this work we derive a theory of thermopiezoelectricity of a body in which the second displacement gradient and the second electric potential gradient are included in the set of independent constitutive variables. This theory may be useful in addressing the above problems, although the number of parameters to be determined is significant and there is no easy way to deduce them from current experiments. We obtain the appropriate thermodynamic restrictions and constitutive equations, with the help of an entropy production inequality proposed by Green and Laws [10]. Next, for both anisotropic and isotropic materials we establish the basic equations of the linear theory and get a uniqueness result for the mixed initial-boundary values problem.

## 2. Basic Equations

We consider a body that at some instant occupies the region *B* of the Euclidean three-dimensional space and is bounded by the piecewise smooth surface ∂B. The motion of the body is referred to the reference configuration *B* and to a fixed system of rectangular Cartesian axes $Ox_{i}$(i=1,2,3).

We shall employ the usual summation and differentiation conventions: Latin subscripts are understood to range over the integers (1,2,3), summation over repeated subscripts is implied and subscripts preceded by a comma denote partial differentiation with respect to the corresponding Cartesian coordinate. In what follows we use a superposed dot to denote partial differentiation with respect to the time *t*. Further, we will neglect the issues of regularity, simply understanding a degree of smoothness sufficient to make sense everywhere.

We restrict our attention to the theory of homogeneous piezoelectric solids. Let $u_{i}$ be the displacement vector, $D_{i}$ the electric displacement vector and $E_{i}$ the electric field vector. The equation of motion is

(1) $$t_{ji,j}+\rho f_{i}=\rho {\ddot{u}}_{i},$$

while the equations for the quasi-static electric field are [35]

(2) $$D_{j,j}=g,\;E_{i}=-\varphi_{,i},$$

where $t_{ij}$ is the stress tensor, $f_{i}$ is the external body force per unit mass, ρ is the reference mass density, *g* is the density of free charges and φ is the electric potential.

Following [14,28,36], we assume that the body is free from initial stress and we postulate an energy balance in the form

$$\begin{array}{cc} & \frac{d}{dt}\int_{P}\rho \left(e+\frac{1}{2}{\dot{u}}_{i}{\dot{u}}_{i}\right)dv=\int_{P}\left(\rho f_{i}{\dot{u}}_{i}+{\dot{D}}_{i}E_{i}+\rho r\right)dv\\ & \;+\int_{\partial P}\left(t_{i}{\dot{u}}_{i}+\mu_{ji}{\dot{u}}_{i,j}+{\dot{Q}}_{i}E_{i}-q\right)da \end{array}$$

for every part *P* of *B* and for every time. Here *e* is the internal energy per unit mass, *r* is the heat supply per unit mass. Moreover, $t_{i}$ is the traction vector, $\mu_{ji}$ is the hypertraction tensor, $Q_{i}$ is the generalized surface charge density and *q* is the heat flux.

Following Ieşan [11], Ieşan [36], for the traction vector, the hyperstress tensor $\mu_{kji}$, the heat flux vector $q_{i}$ and the electric quadrupole $Q_{ji}$ we have

(3) $$t_{i}=t_{ji}n_{j},\;\mu_{ji}=\mu_{kji}n_{k},\;q=q_{i}n_{i},\;{\dot{Q}}_{i}={\dot{Q}}_{ji}n_{j},$$

where $n_{i}$ is the outward unit normal vector to the boundary surface ∂B.

By using Equations (1) and (3) and the divergence theorem, the balance of energy becomes

$$\begin{array}{cc} \int_{P}\rho \dot{e}dv & =\int_{P}[\left(t_{ji}+\mu_{kji,k}\right){\dot{u}}_{i,j}+\mu_{kji}{\dot{u}}_{i,jk}\\ & +\left({\dot{D}}_{i}+{\dot{Q}}_{ji,j}\right)E_{i}+{\dot{Q}}_{ji}E_{i,j}+\rho r-q_{i,i}]dv. \end{array}$$

If we introduce the tensors

(4) $$\tau_{ji}=t_{ji}+\mu_{kji,k},\;\sigma_{i}=D_{i}+Q_{ji,j},$$

Equations (1) and (2)${}_{1}$ can be written as

(5) $$\tau_{ji,j}-\mu_{kji,kj}+\rho f_{i}=\rho {\ddot{u}}_{i},\;\sigma_{i,i}-Q_{ji,ji}=g,$$

and the local form of energy balance becomes

(6) $$\rho \dot{e}=\tau_{ji}{\dot{u}}_{i,j}+\mu_{kji}{\dot{u}}_{i,jk}+{\dot{\sigma }}_{i}E_{i}+{\dot{Q}}_{ji}E_{i,j}+\rho r-q_{i,i}.$$

As proved by Ieşan [11], the invariance of the energy equation for observers in rotating motion the one with respect to the other, leads to

$$\tau_{ji}=\tau_{ij},$$

moreover, without loss of generality, we can suppose

$$\mu_{kji}=\mu_{jki},$$

given that the skew symmetric part makes no contribution to the rate of work over any closed surface in the body, or over the boundary.

Let us set

(7) $$e_{ij}=\frac{1}{2}\left(u_{i,j}+u_{j,i}\right),\;\kappa_{ijk}=u_{k,ij},\;V_{ij}=E_{i,j},$$

then Equation (6) becomes

(8) $$\rho \dot{e}=\tau_{ji}{\dot{e}}_{ij}+\mu_{ijk}{\dot{\kappa }}_{ijk}+{\dot{\sigma }}_{i}E_{i}+{\dot{Q}}_{ji}V_{ij}+\rho r-q_{i,i}.$$

We postulate the entropy production inequality proposed by Green and Laws [10]

$$\frac{d}{dt}\int_{P}\rho \eta dv\geq \int_{P}\frac{\rho r}{\phi }dv-\int_{\partial P}\frac{q}{\phi }da$$

for every part *P* of *B* and every time. Here η is he entropy per unit mass, ϕ is a new strictly positive thermal variable needing a constitutive equation.

Using Equation (3) and the divergence theorem, we have in local form

$$\rho \dot{\eta }\geq \frac{\rho r}{\phi }-{\left(\frac{q_{i}}{\phi }\right)}_{,i}$$

or equivalently

(9) $$\rho \phi \dot{\eta }\geq \rho r-q_{i,i}+\frac{1}{\phi }q_{i}\phi_{,i}.$$

We now introduce the scalar function σ (electric enthalpy) defined by

(10) $$\sigma =\rho \left(e-\phi \eta \right)-\sigma_{i}E_{i}-Q_{ji}V_{ij},$$

and we substitute Equations (8) and (10) in the inequality (9), we then obtain

(11) $$\dot{\sigma }+\rho \dot{\phi }\eta -\tau_{ji}{\dot{e}}_{ij}-\mu_{ijk}{\dot{\kappa }}_{ijk}+\sigma_{i}{\dot{E}}_{i}+Q_{ji}{\dot{V}}_{ij}+\frac{1}{\phi }q_{i}\phi_{,i}\leq 0.$$

## 3. Thermodynamics Restrictions

Let θ be the difference of the absolute temperature *T* and the absolute temperature in the reference configuration $T_{0}$, i.e., $\theta =T-T_{0}$.

We require constitutive equations for σ, ϕ, η, $\tau_{ij}$, $\mu_{ijk}$, $\sigma_{i}$, $Q_{ij}$ and $q_{i}$ and assume that these are functions of the set of variables $S=\left(e_{ij},\kappa_{ijk},E_{i},\theta ,\dot{\theta },\theta_{,i},V_{ij}\right)$

(12) $$\begin{array}{cccccc} & \sigma =\sigma (S),\; & \; & \phi =\phi (S),\; & \; & \eta =\eta (S),\\ \; & \tau_{ij}=\tau_{ij}\left(S\right),\; & \; & \mu_{ijk}=\mu_{ijk}\left(S\right),\; & \; & \sigma_{i}=\sigma_{i}\left(S\right),\\ \; & Q_{ij}=Q_{ij}\left(S\right),\; & \; & q_{i}=q_{i}\left(S\right), \end{array}$$

where $e_{ij}=e_{ji}$, $\kappa_{ijk}=\kappa_{jik}$ e $V_{ij}=V_{ji}$.

If we replace Equation (12) into the inequality (11), we arrive to

$$\begin{array}{cc} & \left[\frac{\partial \sigma }{\partial \theta }+\rho \eta \frac{\partial \phi }{\partial \theta }\right]\dot{\theta }+\left[\frac{\partial \sigma }{\partial \dot{\theta }}+\rho \eta \frac{\partial \phi }{\partial \dot{\theta }}\right]\ddot{\theta }\\ & +\left[\frac{\partial \sigma }{\partial e_{ij}}+\rho \eta \frac{\partial \phi }{\partial e_{ij}}-\tau_{ji}\right]{\dot{e}}_{ij}+\left[\frac{\partial \sigma }{\partial \kappa_{ijk}}+\rho \eta \frac{\partial \phi }{\partial \kappa_{ijk}}-\mu_{ijk}\right]{\dot{\kappa }}_{ijk}\\ & +\left[\frac{\partial \sigma }{\partial \theta_{,i}}+\rho \eta \frac{\partial \phi }{\partial \theta_{,i}}+\frac{1}{\phi }q_{i}\frac{\partial \phi }{\partial \dot{\theta }}\right]{\dot{\theta }}_{,i}+\left[\frac{\partial \sigma }{\partial E_{i}}+\rho \eta \frac{\partial \phi }{\partial E_{i}}+\sigma_{i}\right]\dot{E_{i}}\\ & +\left[\frac{\partial \sigma }{\partial V_{ij}}+\rho \eta \frac{\partial \phi }{\partial V_{ij}}+Q_{ji}\right]{\dot{V}}_{ij}+\frac{1}{\phi }q_{l}[\frac{\partial \phi }{\partial e_{ij}}e_{ij,l}+\frac{\partial \phi }{\partial \kappa_{ijk}}\kappa_{ijk,l}\\ & +\frac{\partial \phi }{\partial \theta }\theta_{,l}+\frac{\partial \phi }{\partial \theta_{,i}}\theta_{,il}+\frac{\partial \phi }{\partial E_{i}}E_{i,l}+\frac{\partial \phi }{\partial V_{ij}}V_{ij,l}]\leq 0. \end{array}$$

We have the following conditions

$$\begin{array}{cccc} q_{l}\frac{\partial \phi }{\partial \kappa_{ijk}} & =0,\; & q_{l}\frac{\partial \phi }{\partial \theta_{,i}}+q_{i}\frac{\partial \phi }{\partial \theta_{,l}} & =0,\\ q_{l}\frac{\partial \phi }{\partial V_{ij}} & =0\; & q_{l}\frac{\partial \phi }{\partial E_{i}}+q_{i}\frac{\partial \phi }{\partial E_{l}} & =0, \end{array}$$

and if we suppose that $q_{i}\neq 0$, we obtain

$$\frac{\partial \phi }{\partial \kappa_{ijk}}=0,\;\frac{\partial \phi }{\partial \theta_{,i}}=0,\;\frac{\partial \phi }{\partial E_{i}}=0,\;\frac{\partial \phi }{\partial V_{ij}}=0,$$

so that ϕ can only depend on $e_{ij}$, θ, $\dot{\theta }$, i.e.,

(13) $$\phi =\phi (e_{ij},\theta ,\dot{\theta }).$$

Consequently, assuming $\partial \phi /\partial \dot{\theta }\neq 0$, we have the following restrictions

(14) $$\begin{array}{cc} \tau_{ij} & =\frac{\partial \sigma }{\partial e_{ij}}+\rho \eta \frac{\partial \phi }{\partial e_{ij}}\\ \mu_{ijk} & =\frac{\partial \sigma }{\partial \kappa_{ijk}}\\ \rho \eta & =-{\left(\frac{\partial \phi }{\partial \dot{\theta }}\right)}^{-1}\frac{\partial \sigma }{\partial \dot{\theta }}\\ q_{i} & =-\phi {\left(\frac{\partial \phi }{\partial \dot{\theta }}\right)}^{-1}\frac{\partial \sigma }{\partial \theta_{,i}}\\ \sigma_{i} & =-\frac{\partial \sigma }{\partial E_{i}},\\ Q_{ji} & =-\frac{\partial \sigma }{\partial V_{ij}} \end{array}$$

and the dissipation inequality is

(15) $$\left(\frac{\partial \sigma }{\partial \theta }+\rho \eta \frac{\partial \phi }{\partial \theta }\right)\dot{\theta }+\frac{1}{\phi }q_{l}\left(\frac{\partial \phi }{\partial \theta }\theta_{,l}+\frac{\partial \phi }{\partial e_{ij}}\kappa_{lij}\right)\leq 0.$$

Taking into account Equations (10), (13) and (14), the equation of energy (8) reduces to

$$\phi \rho \dot{\eta }+q_{i,i}-\rho r+\left(\frac{\partial \sigma }{\partial \theta }+\rho \eta \frac{\partial \phi }{\partial \theta }\right)\dot{\theta }+\frac{\partial \sigma }{\partial \theta_{,i}}{\dot{\theta }}_{,i}=0.$$

## 4. Linear Constitutive Equations

We consider a quadratic Taylor expansion for σ and ϕ with initial point the reference state

(16) $$\begin{array}{cc} \sigma & =\alpha_{ij}^{\left(1\right)}e_{ij}+\alpha_{ijk}^{\left(2\right)}\kappa_{ijk}+\alpha_{i}^{\left(3\right)}E_{i}+\alpha^{\left(4\right)}\theta +\alpha^{\left(5\right)}\dot{\theta }+\alpha_{i}^{\left(6\right)}\theta_{,i}\\ \; & +\alpha_{ij}^{\left(7\right)}V_{ij}+\frac{1}{2}(a_{ijkl}^{\left(11\right)}e_{ij}e_{kl}+a_{ijklhm}^{\left(22\right)}\kappa_{ijk}\kappa_{lhm}+a_{ij}^{\left(33\right)}E_{i}E_{j}\\ \; & +a^{\left(44\right)}\theta^{2}+a^{\left(55\right)}{\dot{\theta }}^{2}+a_{ij}^{\left(66\right)}\theta_{,i}\theta_{,j}+a_{ijkh}^{\left(77\right)}V_{ij}V_{hk})\\ \; & +a_{ijklh}^{\left(12\right)}e_{ij}\kappa_{klh}+a_{ijk}^{\left(13\right)}e_{ij}E_{k}+a_{ij}^{\left(14\right)}e_{ij}\theta +a_{ij}^{\left(15\right)}e_{ij}\dot{\theta }\\ \; & +a_{ijk}^{\left(16\right)}e_{ij}\theta_{,k}+a_{ijkl}^{\left(17\right)}e_{ij}V_{kl}+a_{ijkl}^{\left(23\right)}\kappa_{ijk}E_{l}+a_{ijk}^{\left(24\right)}\kappa_{ijk}\theta \\ \; & +a_{ijk}^{\left(25\right)}\kappa_{ijk}\dot{\theta }+a_{ijkl}^{\left(26\right)}\kappa_{ijk}\theta_{,l}+a_{ijkhl}^{\left(27\right)}\kappa_{ijk}V_{hl}+a_{i}^{\left(34\right)}E_{i}\theta \\ \; & +a_{i}^{\left(35\right)}E_{i}\dot{\theta }+a_{ij}^{\left(36\right)}E_{i}\theta_{,j}+a_{ijk}^{\left(37\right)}E_{i}V_{jk}+a^{\left(45\right)}\theta \dot{\theta }+a_{i}^{\left(46\right)}\theta \theta_{,i}\\ \; & +a_{ij}^{\left(47\right)}\theta V_{ij}+a_{i}^{\left(56\right)}\dot{\theta }\theta_{,i}+a_{ij}^{\left(57\right)}\dot{\theta }V_{ij}+a_{ijk}^{\left(67\right)}\theta_{,i}V_{jk} \end{array}$$

and

(17) $$\begin{array}{cc} \phi & =T_{0}+\beta_{ij}^{\left(1\right)}e_{ij}+\beta^{\left(4\right)}\theta +\beta^{\left(5\right)}\dot{\theta }\\ \; & +\frac{1}{2}\left(b_{ijkl}^{\left(11\right)}e_{ij}e_{kl}+b^{\left(44\right)}\theta^{2}+b^{\left(55\right)}{\dot{\theta }}^{2}\right)\\ \; & +b_{ij}^{\left(14\right)}e_{ij}\theta +b_{ij}^{\left(15\right)}e_{ij}\dot{\theta }+b^{\left(45\right)}\theta \dot{\theta }. \end{array}$$

The coefficients in (16) and (17) satisfy the following symmetry relations

(18) $$\begin{array}{cccc} & \alpha_{ij}^{\left(1\right)}=\alpha_{ji}^{\left(1\right)},\; & \; & \alpha_{ijk}^{\left(2\right)}=\alpha_{jik}^{\left(2\right)},\\ \; & \alpha_{ij}^{\left(7\right)}=\alpha_{ji}^{\left(7\right)}\; & \; & a_{ijkl}^{\left(11\right)}=a_{jikl}^{\left(11\right)}=a_{klij}^{\left(11\right)},\\ \; & a_{ijklhm}^{\left(22\right)}=a_{jiklhm}^{\left(22\right)}=a_{lhmijk}^{\left(22\right)},\; & \; & a_{ij}^{\left(33\right)}=a_{ji}^{\left(33\right)},\\ \; & a_{ij}^{\left(66\right)}=a_{ji}^{\left(66\right)},\; & \; & a_{ijkl}^{\left(77\right)}=a_{jikl}^{\left(77\right)}=a_{klij}^{\left(77\right)},\\ \; & a_{ijklh}^{\left(12\right)}=a_{jiklh}^{\left(12\right)}=a_{ijlkh}^{\left(12\right)},\; & \; & a_{ijk}^{\left(13\right)}=a_{jik}^{\left(13\right)},\\ \; & a_{ij}^{\left(14\right)}=a_{ji}^{\left(14\right)},\; & \; & a_{ij}^{\left(15\right)}=a_{ji}^{\left(15\right)},\\ \; & a_{ijk}^{\left(16\right)}=a_{jik}^{\left(16\right)},\; & \; & a_{ijkl}^{\left(17\right)}=a_{jikl}^{\left(17\right)}=a_{klij}^{\left(17\right)},\\ \; & a_{ijkl}^{\left(23\right)}=a_{jikl}^{\left(23\right)},\; & \; & a_{ijk}^{\left(24\right)}=a_{jik}^{\left(24\right)},\\ \; & a_{ijk}^{\left(25\right)}=a_{jik}^{\left(25\right)},\; & \; & a_{ijkl}^{\left(26\right)}=a_{jikl}^{\left(26\right)},\\ \; & a_{ijkhl}^{\left(27\right)}=a_{jikhl}^{\left(27\right)}=a_{ijklh}^{\left(27\right)},\; & \; & a_{ijk}^{\left(37\right)}=a_{ikj}^{\left(37\right)},\\ \; & a_{ij}^{\left(47\right)}=a_{ji}^{\left(47\right)},\; & \; & a_{ij}^{\left(57\right)}=a_{ji}^{\left(57\right)},\\ \; & a_{ijk}^{\left(67\right)}=a_{ikj}^{\left(67\right)},\\ \; & \beta_{ij}^{\left(1\right)}=\beta_{ji}^{\left(1\right)},\; & \; & b_{ijkl}^{\left(11\right)}=b_{jikl}^{\left(11\right)}=b_{klij}^{\left(11\right)},\\ \; & b_{ij}^{\left(14\right)}=b_{ji}^{\left(14\right)},\; & \; & b_{ij}^{\left(15\right)}=b_{ji}^{\left(15\right)}. \end{array}$$

We suppose that the positive function $\phi (e_{ij},\theta ,\dot{\theta })$ satisfies

$$\phi =\phi \left(e_{ij},\theta ,0\right)=T_{0}+\theta$$

and, consequently, we have

$$\begin{array}{cccccc} & \beta_{ij}^{\left(1\right)}=0,\; & & \beta^{\left(4\right)}=1,\\ & b_{ijkl}^{\left(11\right)}=0,\; & & b^{\left(44\right)}=0,\; & & b_{ij}^{\left(14\right)}=0, \end{array}$$

so that

$$\phi =T_{0}+\theta +\beta \dot{\theta }+\frac{1}{2}b^{\left(55\right)}{\dot{\theta }}^{2}+b_{ij}^{\left(15\right)}e_{ij}\dot{\theta }+b^{\left(45\right)}\theta \dot{\theta }.$$

where, from now on, $\beta =\beta^{\left(5\right)}$.

Assuming that the body in the reference state is free from stress and hyperstress and has zero electric displacement vector, heat flux and electric quadrupole, we obtain

$$\begin{array}{cccccc} & \alpha_{ij}^{\left(1\right)}=0,\; & & \alpha_{ijk}^{\left(2\right)}=0,\\ & \alpha_{i}^{\left(3\right)}=0,\; & & \alpha_{i}^{\left(6\right)}=0,\; & & \alpha_{ij}^{\left(7\right)}=0, \end{array}$$

moreover, linearizing Equation (14) we arrive to

(19) $$\begin{array}{cc} \rho \eta & =-\frac{1}{\beta }(\alpha^{\left(5\right)}+a_{ij}^{\left(15\right)}e_{ij}+a_{ijk}^{\left(25\right)}\kappa_{ijk}+a_{i}^{\left(35\right)}E_{i}\\ \; & +a^{\left(45\right)}\theta +a^{\left(55\right)}\dot{\theta }+a_{i}^{\left(56\right)}\theta_{,i}+a_{ij}^{\left(57\right)}V_{ij})\\ \; & +\frac{\alpha^{\left(5\right)}}{\beta^{2}}\left(b_{ij}^{\left(15\right)}e_{ij}+b^{\left(45\right)}\theta +b^{\left(55\right)}\dot{\theta }\right), \end{array}$$

and

(20) $$\begin{array}{cc} \tau_{ij} & =a_{ijkl}^{\left(11\right)}e_{kl}+a_{ijklh}^{\left(12\right)}\kappa_{klh}+a_{ijk}^{\left(13\right)}E_{k}+a_{ij}^{\left(14\right)}\theta \\ \; & +\left(a_{ij}^{\left(15\right)}-\frac{\alpha^{\left(5\right)}}{\beta }b_{ij}^{\left(15\right)}\right)\dot{\theta }+a_{ijk}^{\left(16\right)}\theta_{,k}+a_{ijkl}^{\left(17\right)}V_{kl},\\ \mu_{ijk} & =a_{hlijk}^{\left(12\right)}e_{hl}+a_{ijkhlm}^{\left(22\right)}\kappa_{hlm}+a_{ijkh}^{\left(23\right)}E_{h}+a_{ijk}^{\left(24\right)}\theta \\ \; & +a_{ijk}^{\left(25\right)}\dot{\theta }+a_{ijkh}^{\left(26\right)}\theta_{,h}+a_{ijklh}^{\left(27\right)}V_{lh},\\ \sigma_{i} & =-(a_{jki}^{\left(13\right)}e_{jk}+a_{jkli}^{\left(23\right)}\kappa_{jkl}+a_{ij}^{\left(33\right)}E_{j}+a_{i}^{\left(34\right)}\theta \\ \; & +a_{i}^{\left(35\right)}\dot{\theta }+a_{ij}^{\left(36\right)}\theta_{,j}+a_{ijk}^{\left(37\right)}V_{jk}),\\ q_{i} & =-\frac{T_{0}}{\beta }(a_{jki}^{\left(16\right)}e_{jk}+a_{jkli}^{\left(26\right)}\kappa_{jkl}+a_{ji}^{\left(36\right)}E_{j}+a_{i}^{\left(46\right)}\theta \\ \; & +a_{i}^{\left(56\right)}\dot{\theta }+a_{ij}^{\left(66\right)}\theta_{,j}+a_{ijk}^{\left(67\right)}V_{jk}),\\ Q_{ij} & =-(a_{klij}^{\left(17\right)}e_{kl}+a_{klmij}^{\left(27\right)}\kappa_{klm}+a_{kij}^{\left(37\right)}E_{k}+a_{ij}^{\left(47\right)}\theta \\ \; & +a_{ij}^{\left(57\right)}\dot{\theta }+a_{kij}^{\left(67\right)}\theta_{,k}+a_{ijkl}^{\left(77\right)}V_{kl}). \end{array}$$

Linearizing the coefficients of the entropy inequality (15) we obtain

$$A\left(S\right)\dot{\theta }+B_{i}\left(S\right)\theta_{,i}\leq 0$$

where

$$\begin{array}{cc} A(S) & =\alpha^{\left(4\right)}+a_{ij}^{\left(14\right)}e_{ij}+a_{ijk}^{\left(24\right)}\kappa_{ijk}+a_{i}^{\left(34\right)}E_{i}\\ & +a^{\left(44\right)}\theta +a^{\left(45\right)}\dot{\theta }+a_{i}^{\left(46\right)}\theta_{,i}+a_{ij}^{\left(47\right)}V_{ij}\\ & -\frac{1}{\beta }(\alpha^{\left(5\right)}+a_{ij}^{\left(15\right)}e_{ij}+a_{ijk}^{\left(25\right)}\kappa_{ijk}+a_{i}^{\left(35\right)}E_{i}\\ & +a^{\left(45\right)}\theta +a^{\left(55\right)}\dot{\theta }+a_{i}^{\left(56\right)}\theta_{,i}+a_{ij}^{\left(57\right)}V_{ij})\\ & +\frac{\alpha^{\left(5\right)}}{\beta^{2}}\left(b_{ij}^{\left(15\right)}e_{ij}+b^{\left(45\right)}\left(\theta -\beta \dot{\theta }\right)+b^{\left(55\right)}\dot{\theta }\right),\\ B_{i}\left(S\right) & =-\frac{1}{\beta }(a_{jki}^{\left(16\right)}e_{jk}+a_{jkli}^{\left(26\right)}\kappa_{jkl}+a_{ji}^{\left(36\right)}E_{j}+a_{i}^{\left(46\right)}\theta \\ & +a_{i}^{\left(56\right)}\dot{\theta }+a_{ij}^{\left(66\right)}\theta_{,j}+a_{ijk}^{\left(67\right)}V_{jk}). \end{array}$$

If we define

$$S_{0}=S{|}_{\dot{\theta }=0,\theta_{,i}=0}=\left(e_{ij},\kappa_{ijk},E_{i},\theta ,0,0,V_{ij}\right)$$

by virtue of the dissipation inequality we arrive to

$$A\left(S_{0}\right)=0,\;B_{i}\left(S_{0}\right)=0.$$

From the first condition we have

$$\alpha^{\left(5\right)}=\beta \alpha^{\left(4\right)}$$

and

$$\begin{array}{cccc} a_{ij}^{\left(15\right)} & =\beta a_{ij}^{\left(14\right)}+\alpha^{\left(4\right)}b_{ij}^{\left(15\right)},\\ a_{ijk}^{\left(25\right)} & =\beta a_{ijk}^{\left(24\right)},\; & a_{i}^{\left(35\right)} & =\beta a_{i}^{\left(34\right)},\;\\ a^{\left(45\right)} & =\beta a^{\left(44\right)}+\alpha^{\left(4\right)}b^{\left(45\right)},\; & a_{ij}^{\left(57\right)} & =\beta a_{ij}^{\left(47\right)}, \end{array}$$

while from the second one we obtain

$$\begin{array}{cccccc} a_{ijk}^{\left(16\right)} & =0,\; & a_{ijkl}^{\left(26\right)} & =0,\; & a_{ij}^{\left(36\right)} & =0,\\ a_{i}^{\left(46\right)} & =0,\; & a_{ijk}^{\left(67\right)} & =0. \end{array}$$

Finally we have

$$\begin{array}{cc} A(S) & =-\frac{1}{\beta }\left(\gamma \dot{\theta }+a_{i}^{\left(56\right)}\theta_{,i}\right),\\ B_{i}\left(S\right) & =-\frac{1}{\beta }\left(a_{i}^{\left(56\right)}\dot{\theta }+a_{ij}^{\left(66\right)}\theta_{,j}\right), \end{array}$$

where we defined

$$\gamma =a^{\left(55\right)}-\beta^{2}a^{\left(44\right)}-\alpha^{\left(4\right)}b^{\left(55\right)}.$$

Using these relations, the constitutive Equations (19) and (20) can be expressed as

(21) $$\begin{array}{cc} \tau_{ij} & =a_{ijkl}^{\left(11\right)}e_{kl}+a_{ijklh}^{\left(12\right)}\kappa_{klh}+a_{ijk}^{\left(13\right)}E_{k}\\ \; & +a_{ij}^{\left(14\right)}\left(\theta +\beta \dot{\theta }\right)+a_{ijkl}^{\left(17\right)}V_{kl},\\ \mu_{ijk} & =a_{lhijk}^{\left(12\right)}e_{lh}+a_{ijklhm}^{\left(22\right)}\kappa_{lhm}+a_{ijkl}^{\left(23\right)}E_{l}\\ \; & +a_{ijk}^{\left(24\right)}\left(\theta +\beta \dot{\theta }\right)+a_{ijklh}^{\left(27\right)}V_{lh},\\ -\sigma_{i} & =a_{jki}^{\left(13\right)}e_{jk}+a_{jkli}^{\left(23\right)}\kappa_{jkl}+a_{ij}^{\left(33\right)}E_{j}\\ \; & +a_{i}^{\left(34\right)}\left(\theta +\beta \dot{\theta }\right)+a_{ijk}^{\left(37\right)}V_{jk},\\ -\rho \eta & =a_{ij}^{\left(14\right)}e_{ij}+a_{ijk}^{\left(24\right)}\kappa_{ijk}+a_{i}^{\left(34\right)}E_{i}\\ \; & +a^{\left(44\right)}\left(\theta +\beta \dot{\theta }\right)+a_{ij}^{\left(47\right)}V_{ij}\\ \; & +\frac{1}{\beta }\left(\gamma \dot{\theta }+a_{i}^{\left(56\right)}\theta_{,i}\right)+\alpha^{\left(4\right)},\\ -Q_{ij} & =a_{klij}^{\left(17\right)}e_{kl}+a_{klmij}^{\left(27\right)}\kappa_{klm}+a_{kij}^{\left(37\right)}E_{k}\\ \; & +a_{ij}^{\left(47\right)}\left(\theta +\beta \dot{\theta }\right)+a_{ijkl}^{\left(77\right)}V_{kl},\\ -\frac{q_{i}}{T_{0}} & =\frac{1}{\beta }\left(a_{i}^{\left(56\right)}\dot{\theta }+a_{ij}^{\left(66\right)}\theta_{,j}\right). \end{array}$$

In the linear approximation, the energy equation is

(22) $$\rho T_{0}\dot{\eta }+q_{i,i}-\rho r=0$$

or, with help of Equation (21), we arrive to

$$\begin{array}{cc} \; & a_{ij}^{\left(14\right)}{\dot{e}}_{ij}+a_{ijk}^{\left(24\right)}{\dot{\kappa }}_{ijk}+a_{i}^{\left(34\right)}{\dot{E}}_{i}+a^{\left(44\right)}\left(\dot{\theta }+\beta \ddot{\theta }\right)+a_{ij}^{\left(47\right)}{\dot{V}}_{ij}\\ \; & \;+\frac{1}{\beta }\left(\gamma \ddot{\theta }+2a_{i}^{\left(56\right)}{\dot{\theta }}_{,i}+a_{ij}^{\left(66\right)}\theta_{,ij}\right)+\frac{1}{T_{0}}\rho r=0. \end{array}$$

The dissipation inequality (15) becomes

$$\frac{1}{\beta }\left(\gamma {\dot{\theta }}^{2}+2a_{i}^{\left(56\right)}\dot{\theta }\theta_{,i}+a_{ij}^{\left(66\right)}\theta_{,i}\theta_{,j}\right)\geq 0.$$

The following quadratic form is defined from the previous dissipation inequality

(23) $$P\left(\xi ,\eta_{i}\right)=\frac{1}{\beta }\left(\gamma \xi^{2}+2a_{i}^{\left(56\right)}\xi \eta_{i}+a_{ij}^{\left(66\right)}\eta_{i}\eta_{j}\right)$$

so that

(24) $$P\left(\xi ,\eta_{i}\right)\geq 0,\;\forall \xi ,\eta_{i}.$$

If we consider the symmetric matrix associated with the quadratic form P

$$\frac{1}{\beta }\left(\begin{array}{cc} \gamma & a_{j}^{\left(56\right)}\\ a_{i}^{\left(56\right)} & a_{ij}^{\left(66\right)} \end{array}\right),$$

its positive semi-definiteness implies, in particular, that

$$\frac{\gamma }{\beta }\geq 0,\;\frac{1}{\beta }a_{ij}^{\left(66\right)}\eta_{i}\eta_{j}\geq 0,\;\forall \eta_{i}.$$

The basic equations of linear theory of thermopiezoelectric solids consist of the equations of motion (5), the equation of energy (22), the geometrical Equations (7) and (2)${}_{2}$, the constitutive Equation (21) with the restriction (24), on B×I, where $I=[0,t_{0})$, where $t_{0}\leq +\infty$.

Following Toupin [14] and Mindlin [15], we consider $P_{i}$, $R_{i}$, Λ and *H* defined in such a way that the total rate of work of the surface forces over the smooth surface ∂P can be expressed in the form

$$\begin{array}{cc} & \int_{\partial P}\left(t_{ki}{\dot{u}}_{i}+\mu_{kji}{\dot{u}}_{i,j}-\varphi {\dot{D}}_{k}-\varphi_{,i}{\dot{Q}}_{ki}\right)n_{k}\;dA\\ & \;=\int_{\partial P}\left(P_{i}{\dot{u}}_{i}+R_{i}D{\dot{u}}_{i}-\varphi \dot{\Lambda }-\dot{H}D\varphi \right)dA. \end{array}$$

Here we used

$$\begin{array}{cc} & P_{i}=\left(\tau_{ji}-\mu_{kji,k}\right)n_{j}-D_{j}\left(\mu_{kji}n_{k}\right)+\left(D_{l}n_{l}\right)\mu_{kji}n_{k}\;n_{j}\\ & \Lambda =\left(\sigma_{j}-Q_{kj,k}\right)n_{j}-D_{j}\left(Q_{kj}n_{k}\right)+\left(D_{l}n_{l}\right)Q_{kj}n_{k}n_{j}, \end{array}$$

and

$$R_{i}=\mu_{kji}n_{k}n_{j},\;H=Q_{kj}n_{k}n_{j},$$

where $D\equiv n_{i}\partial /\partial x_{i}$ is the normal derivative and

$$D_{i}\equiv \left(\delta_{ij}-n_{i}n_{j}\right)\frac{\partial }{\partial x_{j}}$$

is the surface gradient.

Now, we denote with

$$U=(u_{i},\theta ,\varphi )$$

the solutions of the mixed initial-boundary value problem Π defined by Equations (5), (22), (7), (2)${}_{2}$ and (21) and the following initial conditions

$$\begin{array}{cccc} u_{i}\left(0\right) & =u_{i}^{0},\; & {\dot{u}}_{i}\left(0\right) & =v_{i}^{0},\\ \theta (0) & =\theta^{0},\; & \eta (0) & =\eta^{0}, \end{array}$$

in $\bar{B}$ and the following boundary conditions

$$\begin{array}{cccc} u_{i} & ={\hat{u}}_{i}\;\mathrm{on}\;S_{1}\times I,\; & P_{i} & ={\hat{P}}_{i}\;\mathrm{on}\;\Sigma_{1}\times I,\\ Du_{i} & ={\hat{d}}_{i}\;\mathrm{on}\;S_{2}\times I,\; & R_{i} & ={\hat{R}}_{i}\;\mathrm{on}\;\Sigma_{2}\times I,\\ \theta & =\hat{\theta }\;\mathrm{on}\;S_{3}\times I,\; & q_{i}n_{i} & =\hat{q}\;\mathrm{on}\;\Sigma_{3}\times I,\\ \varphi & =\hat{\varphi }\;\mathrm{on}\;S_{4}\times I,\; & \Lambda & =\hat{\Lambda }\;\mathrm{on}\;\Sigma_{4}\times I,\\ D\varphi & =\hat{\xi }\;\mathrm{on}\;S_{5}\times I,\; & H & =\hat{H}\;\mathrm{on}\;\Sigma_{5}\times I, \end{array}$$

with $u_{i}^{0}$, $v_{i}^{0}$, $\theta^{0}$, $\eta^{0}$, ${\hat{u}}_{i}$, ${\hat{d}}_{i}$, $\hat{\theta }$, $\hat{\varphi }$, $\hat{\xi }$, ${\hat{P}}_{i}$, ${\hat{R}}_{i}$, $\hat{q}$, $\hat{\Lambda }$ and $\hat{H}$ are prescribed functions and the surfaces $S_{i}$ and $\Sigma_{i}$ are such that

$${\bar{S}}_{i}\cup \Sigma_{i}=\partial B\;\;S_{i}\cap \Sigma_{i}=⌀,\;i=1,\ldots ,5$$

where the closure is relative to ∂B. The (external) data of the mixed initial-boundary value problem in concern are

$$\Gamma =\left\{f_{i},g,r,u_{i}^{0},v_{i}^{0},\theta^{0},\eta^{0},{\hat{u}}_{i},{\hat{d}}_{i},\hat{\theta },\hat{\varphi },\hat{\xi },{\hat{P}}_{i},{\hat{R}}_{i},\hat{q},\hat{\Lambda },\hat{H}\right\}.$$

## 5. A Uniqueness Result

In this section, we establish a uniqueness result for a initial-boundary value problem Π. To this aim, using the constitutive Equation (21) we prove that

(25) $$\begin{array}{cc} \; & \tau_{ij}{\dot{e}}_{ij}+\mu_{ijk}{\dot{\kappa }}_{ijk}+{\dot{\sigma }}_{i}E_{i}+{\dot{Q}}_{ji}V_{ij}+\rho \dot{\eta }\left(\theta +\beta \dot{\theta }\right)=\\ \; & \;=\dot{W}+\dot{F}-\frac{1}{2}a^{\left(44\right)}\frac{d}{dt}{\left(\theta +\beta \dot{\theta }\right)}^{2}\\ \; & \;-\frac{1}{\beta }\left(\gamma \ddot{\theta }+a_{i}^{\left(56\right)}{\dot{\theta }}_{,i}\right)\left(\theta +\beta \dot{\theta }\right), \end{array}$$

where *W* is the following quadratic form in the strain measures $e_{ij}$ and $\kappa_{ijk}$

$$W=\frac{1}{2}a_{ijkl}^{\left(11\right)}e_{ij}e_{kl}+\frac{1}{2}a_{ijklhm}^{\left(22\right)}\kappa_{ijk}\kappa_{lhm}+a_{ijklh}^{\left(12\right)}e_{ij}\kappa_{klh}$$

and *F* is a quadratic form in the variables $E_{i}$, $V_{ij}$, $\theta +\beta \dot{\theta }$

$$\begin{array}{cc} F & =-\frac{1}{2}a_{ij}^{\left(33\right)}E_{i}E_{j}-\frac{1}{2}a_{ijkl}^{\left(77\right)}V_{ij}V_{kl}-a_{ijk}^{\left(37\right)}E_{i}V_{jk}\\ & \;-a_{i}^{\left(34\right)}E_{i}\left(\theta +\beta \dot{\theta }\right)-a_{ij}^{\left(47\right)}V_{ij}\left(\theta +\beta \dot{\theta }\right). \end{array}$$

On the other hand, taking into account Equation (2)${}_{2}$, (4), (5), (21) and (22), we have

(26) $$\begin{array}{cc} \; & \tau_{ij}{\dot{e}}_{ij}+\mu_{ijk}{\dot{\kappa }}_{ijk}+{\dot{\sigma }}_{i}E_{i}+{\dot{Q}}_{ji}V_{ij}+\rho \dot{\eta }\left(\theta +\beta \dot{\theta }\right)=\\ \; & \;={\left[t_{ki}{\dot{u}}_{i}+\mu_{jki}{\dot{u}}_{i,j}-{\dot{D}}_{k}\varphi -{\dot{Q}}_{kj}\varphi_{,j}-\frac{q_{k}}{T_{0}}\left(\theta +\beta \dot{\theta }\right)\right]}_{,k}\\ \; & \;+\rho f_{i}{\dot{u}}_{i}-\dot{g}\varphi +\frac{\rho r}{T_{0}}\left(\theta +\beta \dot{\theta }\right)-\rho {\ddot{u}}_{i}{\dot{u}}_{i}\\ \; & \;-\frac{1}{\beta }\left(\theta_{,k}+\beta {\dot{\theta }}_{,k}\right)\left(a_{k}^{\left(56\right)}\dot{\theta }+a_{kj}^{\left(66\right)}\theta_{,j}\right) \end{array}$$

Equations (25) and (26) imply

(27) $$\begin{array}{cc} \; & \frac{d}{dt}\left[W+F+\frac{1}{2}\rho {\dot{u}}_{i}{\dot{u}}_{i}-\frac{1}{2}a^{\left(44\right)}{\left(\theta +\beta \dot{\theta }\right)}^{2}\right]\\ \; & \;-{\left[t_{ki}{\dot{u}}_{i}+\mu_{jki}{\dot{u}}_{i,j}-{\dot{D}}_{k}\varphi -{\dot{Q}}_{kj}\varphi_{,j}-\frac{q_{k}}{T_{0}}\left(\theta +\beta \dot{\theta }\right)\right]}_{,k}\\ \; & \;-\left[\rho f_{i}{\dot{u}}_{i}-\dot{g}\varphi +\frac{\rho r}{T_{0}}\left(\theta +\beta \dot{\theta }\right)\right]=\\ \; & \;=-\frac{1}{\beta }\left(\theta_{,k}+\beta {\dot{\theta }}_{,k}\right)\left(a_{k}^{\left(56\right)}\dot{\theta }+a_{kj}^{\left(66\right)}\theta_{,j}\right)\\ \; & \;+\frac{1}{\beta }\left(\gamma \ddot{\theta }+a_{i}^{\left(56\right)}{\dot{\theta }}_{,i}\right)\left(\theta +\beta \dot{\theta }\right) \end{array}$$

It is easy to see that

(28) $$\begin{array}{cc} \; & \frac{1}{\beta }\left(\gamma \ddot{\theta }+a_{i}^{\left(56\right)}{\dot{\theta }}_{,i}\right)\left(\theta +\beta \dot{\theta }\right)\\ \; & \;-\frac{1}{\beta }\left(\theta_{,k}+\beta {\dot{\theta }}_{,k}\right)\left(a_{k}^{\left(56\right)}\dot{\theta }+a_{kj}^{\left(66\right)}\theta_{,j}\right)\\ \; & \;=-P\left(\dot{\theta ,}\theta_{,i}\right)+\frac{1}{2}\frac{\gamma }{\beta^{2}}\frac{d}{dt}{\left(\theta +\beta \dot{\theta }\right)}^{2}-\frac{1}{2}\beta \frac{d}{dt}P\left(-\frac{\theta }{\beta },\theta_{,i}\right) \end{array}$$

with P defined by (23) and satisfying (24).

Taking into account Equations (27) and (28) and introducing the following quadratic form in the variable $E_{i}$, $V_{ij}$, $\theta +\beta \dot{\theta }$

$$G=F-\frac{1}{2}\left(a^{\left(44\right)}+\frac{\gamma }{\beta^{2}}\right){\left(\theta +\beta \dot{\theta }\right)}^{2}$$

we can write

(29) $$\begin{array}{cc} \; & \frac{d}{dt}\left[E+\frac{1}{2}\beta P\left(-\frac{\theta }{\beta },\theta_{,i}\right)\right]+\\ \; & \;-{\left[t_{ki}{\dot{u}}_{i}+\mu_{jki}{\dot{u}}_{i,j}-{\dot{D}}_{k}\varphi -{\dot{Q}}_{kj}\varphi_{,j}-\frac{q_{k}}{T_{0}}\left(\theta +\beta \dot{\theta }\right)\right]}_{,k}\\ \; & \;-\left[\rho f_{i}{\dot{u}}_{i}-\dot{g}\varphi +\frac{\rho r}{T_{0}}\left(\theta +\beta \dot{\theta }\right)\right]\\ \; & \;=-P(\dot{\theta ,}\theta_{,i})\leq 0 \end{array}$$

where

$$E=W+G+\frac{1}{2}\rho {\dot{u}}_{i}{\dot{u}}_{i}.$$

It easily follows the next theorem

**Theorem** **1** (Uniqueness). *Assume that*

*(i)*  *ρ, β, $T_{0}>0$,*
*(ii)*  *the constitutive coefficients satisfy the relations (18),*
*(iii)*  *W is a positive semi-definite quadratic form,*
*(iv)*  *G is a positive definite quadratic form.*

*Then, if $S_{4}$ is nonempty, the initial-boundary values problem *Π* has at most one solution.*

**Proof.**  Suppose that we have two solutions of the problem Π. Then their difference $\left(u_{i},\theta ,\varphi \right)$ corresponds to null data. By integrating Equation (29), we have

$$\frac{d}{dt}\int_{B}\left[E+\frac{1}{2}\beta P\left(-\frac{\theta }{\beta },\theta_{,i}\right)\right]\leq 0.$$

The last integral must be a decreasing function with respect to time, but since it is not negative and initially null, it can be deduced that

(30) $${\dot{u}}_{i}=0,\;\theta +\beta \dot{\theta }=0,\;E_{i}=0\;\mathrm{on}\;B\times I.$$

Taking into account that Equation (30)${}_{1,2}$ are linear homogeneous equations with null initial conditions and using Equation (2)${}_{2}$,we conclude that

$$u_{i}=0,\;\theta =0,\;\varphi =\mathrm{const}\;\mathrm{on}\;B\times I.$$

If $S_{4}\neq ⌀$ we obtain the uniqueness result, in fact it is

$$\varphi =0\;\mathrm{on}\;S_{4}\times I\;\Rightarrow \;\varphi =0\;\mathrm{on}\;B\times I.$$

□

## 6. Isotropic Thermoelastic Materials

For the class of isotropic materials with a center of symmetry, the constitutive coefficients become

$$\begin{array}{cc} & a_{ijkmnr}^{\left(22\right)}=\\ & =\gamma_{1}\left(\delta_{ij}\delta_{km}\delta_{nr}+\delta_{ij}\delta_{kn}\delta_{mr}+\delta_{ik}\delta_{jr}\delta_{mn}+\delta_{ir}\delta_{jk}\delta_{mn}\right)\\ & \;+\gamma_{2}\left(\delta_{ik}\delta_{jm}\delta_{nr}+\delta_{ik}\delta_{jn}\delta_{mr}+\delta_{im}\delta_{jk}\delta_{nr}+\delta_{in}\delta_{jk}\delta_{mr}\right)\\ & \;+\gamma_{3}\delta_{ij}\delta_{kr}\delta_{mn}+\gamma_{4}\left(\delta_{im}\delta_{jn}\delta_{kr}+\delta_{in}\delta_{jm}\delta_{kr}\right)\\ & \;+\gamma_{5}\left(\delta_{im}\delta_{jr}\delta_{kn}+\delta_{in}\delta_{jr}\delta_{km}+\delta_{ir}\delta_{jm}\delta_{kn}+\delta_{ir}\delta_{jn}\delta_{km}\right),\\ & a_{ijkl}^{\left(11\right)}=\lambda \delta_{ij}\delta_{kl}+\mu \left(\delta_{ik}\delta_{jl}+\delta_{il}\delta_{jk}\right),\\ & a_{ijkl}^{\left(17\right)}=\lambda^{*}\delta_{ij}\delta_{kl}+\mu^{*}\left(\delta_{ik}\delta_{jl}+\delta_{il}\delta_{jk}\right),\\ & a_{ijkl}^{\left(23\right)}=\alpha^{0}\delta_{ij}\delta_{kl}+\beta^{0}\left(\delta_{ik}\delta_{jl}+\delta_{il}\delta_{jk}\right),\\ & a_{ijkl}^{\left(77\right)}=\tilde{\lambda }\delta_{ij}\delta_{kl}+\tilde{\mu }\left(\delta_{ik}\delta_{jl}+\delta_{il}\delta_{jk}\right),\\ & a_{ij}^{\left(14\right)}=\alpha^{\left(14\right)}\delta_{ij},\;a_{ij}^{\left(33\right)}=\alpha^{\left(33\right)}\delta_{ij},\\ & a_{ij}^{\left(47\right)}=\alpha^{\left(47\right)}\delta_{ij},\;a_{ij}^{\left(66\right)}=\alpha^{\left(66\right)}\delta_{ij}, \end{array}$$

and

$$\begin{array}{cccccccc} & a_{ijklh}^{\left(12\right)}=0, & \; & a_{ijk}^{\left(13\right)}=0, & \; & a_{ijk}^{\left(24\right)}=0,\\ & a_{ijklh}^{\left(27\right)}=0, & \; & a_{i}^{\left(34\right)}=0, & \; & a_{ijk}^{\left(37\right)}=0, & \; & a_{i}^{\left(56\right)}=0. \end{array}$$

Consequently, the constitutive Equation (21) reduce to

$$\begin{array}{cc} \tau_{ij} & =\lambda e_{kk}\delta_{ij}+2\mu e_{ij}+\alpha^{\left(14\right)}\delta_{ij}\left(\theta +\beta \dot{\theta }\right)\\ & +\lambda^{*}V_{kk}\delta_{ij}+2\mu^{*}V_{ij},\\ \mu_{ijk} & =\gamma_{1}\left(\kappa_{hhi}\delta_{jk}+2\kappa_{khh}\delta_{ij}+\kappa_{hhj}\delta_{ik}\right)+2\gamma_{2}(\kappa_{ihh}\delta_{jk}\\ & +\kappa_{jhh}\delta_{ik})+\gamma_{3}\kappa_{hhk}\delta_{ij}+2\gamma_{4}\kappa_{ijk}+2\gamma_{5}\left(\kappa_{kji}+\kappa_{kij}\right)\\ & +\alpha_{0}\delta_{ij}E_{k}+\beta_{0}\left(\delta_{ik}E_{j}+\delta_{jk}E_{i}\right),\\ -\sigma_{i} & =\alpha_{0}\kappa_{kki}+2\beta_{0}\kappa_{ikk}+\alpha^{\left(33\right)}E_{i},\\ -\rho \eta & =\alpha^{\left(14\right)}e_{kk}+a^{\left(44\right)}\left(\theta +\beta \dot{\theta }\right)+\alpha^{\left(47\right)}V_{kk}+\\ & +\frac{1}{\beta }\gamma \dot{\theta }+\alpha^{\left(4\right)}\\ -Q_{ij} & =\lambda^{*}e_{kk}\delta_{ij}+2\mu^{*}e_{ij}+\alpha^{\left(47\right)}\delta_{ij}\left(\theta +\beta \dot{\theta }\right)\\ & +\tilde{\lambda }V_{kk}\delta_{ij}+2\tilde{\mu }V_{ij},\\ -\frac{q_{i}}{T_{0}} & =\frac{1}{\beta }\alpha^{\left(66\right)}\theta_{,i}. \end{array}$$

Using these equations and (2)${}_{2}$, Equations (5) and (22) can be written as follows

$$\begin{array}{cc} \; & \mu u_{i,jj}+\left(\lambda +\mu \right)u_{j,ji}+\alpha^{\left(14\right)}\left(\theta_{,i}+\beta {\dot{\theta }}_{,i}\right)-\left(\lambda^{*}+2\mu^{*}\right)\varphi_{,ijj}\\ \; & \;-4\left(\gamma_{1}+\gamma_{2}+\gamma_{5}\right)u_{j,jikk}-\left(\gamma_{3}+2\gamma_{4}\right)u_{i,jjkk}\\ \; & \;-\left(\alpha_{0}+2\beta_{0}\right)\varphi_{,ijj}+\rho f_{i}=\rho {\ddot{u}}_{i},\\ \; & \left(\lambda^{*}+2\mu^{*}-\alpha_{0}-2\beta_{0}\right)u_{j,jkk}+\alpha^{\left(33\right)}\varphi_{,jj}\\ \; & \;+\alpha^{\left(47\right)}\left(\theta_{,jj}+\beta {\dot{\theta }}_{,jj}\right)-\left(\tilde{\lambda }+2\tilde{\mu }\right)\varphi_{,jjkk}=g,\\ \; & \alpha^{\left(14\right)}{\dot{u}}_{j,j}+a^{\left(44\right)}\dot{\theta }+\left(a^{\left(44\right)}+\frac{1}{\beta^{2}}\gamma \right)\beta \ddot{\theta }\\ \; & \;+\frac{1}{\beta }\alpha^{\left(66\right)}\theta_{,jj}+\frac{1}{T_{0}}\rho r=0. \end{array}$$

Now, we remark that the condition (24) of positive semi-definiteness of quadratic form P is equivalent to

$$\frac{\gamma }{\beta }\geq 0,\;\frac{\alpha^{\left(66\right)}}{\beta }\geq 0.$$

In particular, when β>0 Equation (24) is equivalent to γ≥0, $\alpha^{\left(66\right)}\geq 0$.

In the isotropic case, the quadratic form *W* can be expressed as the sum of two independent quadratic forms, the first one $W_{1}$ in the variables $e_{ij}$ and the second one $W_{2}$ in the variables $\kappa_{ijk}$.

The positive semi-definiteness of $W_{1}$ leads to the well known conditions

(31) μ≥0,3λ+2μ≥0.

Regarding $W_{2}$, if we choose the following ordering of the variables:

$$\begin{array}{cc} \{ & \kappa_{221},\kappa_{331},\kappa_{111},\kappa_{122},\kappa_{133},\\ & \kappa_{332},\kappa_{112},\kappa_{222},\kappa_{233},\kappa_{211},\\ & \kappa_{113},\kappa_{223},\kappa_{333},\kappa_{311},\kappa_{322},\\ & \kappa_{123},\kappa_{231},\kappa_{312}\} \end{array}$$

the corresponding matrix is of type

$$\left(\begin{array}{cccc} A_{5} & 0 & 0 & 0\\ 0 & A_{5} & 0 & 0\\ 0 & 0 & A_{5} & 0\\ 0 & 0 & 0 & A_{3} \end{array}\right)$$

where $A_{5}$ is a 5×5 matrix and $A_{3}$ is a 3×3 matrix. The eigenvalues of $A_{3}$ are

$$\begin{array}{cccc} & 2(\gamma_{4}-\gamma_{5}),\; & & \mathrm{multiplicity}\;2\\ & 2(\gamma_{4}+2\gamma_{5}),\; & & \mathrm{multiplicity}\;1 \end{array}$$

The matrix $A_{5}$ is similar to

$$\left(\begin{array}{ccccc} \gamma_{3}+\gamma_{4} & \frac{1}{2}\left(2\gamma_{1}+\gamma_{3}\right) & 2(\gamma_{1}+\gamma_{5}) & 0 & 0\\ 2\gamma_{1}+\gamma_{3} & \xi & 2(\gamma_{1}+2\gamma_{2}) & 0 & 0\\ 2(\gamma_{1}+\gamma_{5}) & \gamma_{1}+2\gamma_{2} & 2(2\gamma_{2}+\gamma_{4}+\gamma_{5}) & 0 & 0\\ 0 & 0 & 0 & \xi_{1} & 0\\ 0 & 0 & 0 & 0 & \xi_{2} \end{array}\right)$$

where

$$\xi =2\left(\gamma_{1}+\gamma_{2}+\gamma_{5}\right)+\frac{1}{2}\left(\gamma_{3}+2\gamma_{4}\right)$$

and

$$\xi_{1,2}=\frac{1}{2}\left(3\gamma_{4}+2\gamma_{5}\pm \sqrt{{\left(\gamma_{4}+2\gamma_{5}\right)}^{2}+16\gamma_{5}^{2}}\right)$$

Consequently, the conditions of positive semi-definiteness of quadratic form $W_{2}$ are

(32) $$\begin{array}{c} \begin{array}{ccc} \gamma_{3}+\gamma_{4}\geq 0, & \; & \;\gamma_{4}-\gamma_{5}\geq 0,\;\gamma_{4}+2\gamma_{5}\geq 0,\\ 2\gamma_{2}+\gamma_{4}+\gamma_{5}\geq 0, & \; & \;4\left(\gamma_{1}+\gamma_{2}+\gamma_{5}\right)+\left(\gamma_{3}+2\gamma_{4}\right)\geq 0, \end{array}\\ \left(\gamma_{3}+\gamma_{4}\right)\left(4\gamma_{2}+3\gamma_{4}+4\gamma_{5}\right)\geq {\left(2\gamma_{1}-\gamma_{4}\right)}^{2},\\ \left(\gamma_{3}+\gamma_{4}\right)\left(2\gamma_{2}+\gamma_{4}+\gamma_{5}\right)\geq 2{\left(\gamma_{1}+\gamma_{5}\right)}^{2},\\ \left(2\gamma_{2}+\gamma_{4}+\gamma_{5}\right)\left(\gamma_{3}+4\gamma_{4}+6\gamma_{5}\right)\geq 2{\left(\gamma_{1}-\gamma_{4}-\gamma_{5}\right)}^{2},\\ \left(\gamma_{4}+2\gamma_{5}\right)[\left(5\gamma_{3}+4\gamma_{4}-2\gamma_{5}\right)\left(10\gamma_{2}+3\gamma_{4}+\gamma_{5}\right)\\ \;-2{\left(5\gamma_{1}-\gamma_{4}+3\gamma_{5}\right)}^{2}]\geq 0. \end{array}$$

On the other hand, the quadratic form *G* is positive definite if and only if

(33) $$\begin{array}{cc} \; & \begin{array}{cccc} \; & \tilde{\mu }<0,\; & \; & 3\tilde{\lambda }+2\tilde{\mu }<0,\\ \; & \alpha^{\left(33\right)}<0,\; & \; & a^{\left(44\right)}+\frac{\gamma }{\beta^{2}}<0, \end{array}\\ \; & \left(3\tilde{\lambda }+2\tilde{\mu }\right)\left(a^{\left(44\right)}+\frac{\gamma }{\beta^{2}}\right)>3{\left(\alpha^{\left(47\right)}\right)}^{2}. \end{array}$$

For isotropic materials we obtain the following uniqueness result for the mixed initial-boundary values problem

**Theorem** **2.** *Let us assume that*

*(i)*   *ρ>0, $T_{0}>0$, β>0, γ≥0, $a^{\left(66\right)}\geq 0$,*
*(ii)*  *the inequalities* (31), (32) *and* (33) *hold*.

*If $S_{4}$ is nonempty, the initial-boundary values problem considered has at most one solution.*

## 7. Conclusions and Further Developments

In this manuscript:

- We have derived a theory of thermopiezoelectricity of a body in which the second displacement gradient and the second electric potential gradient are included in the set of independent constitutive variables;
- We have obtained appropriate thermodynamic restrictions and constitutive equations, with the help of the entropy production inequality proposed by Green and Laws [10];
- We have established the balance equations and constitutive equations of linear theory;
- We have set the mixed initial-boundary values problem for this theory and have achieved a result of uniqueness for this problem;
- We then established the balance equations and the constitutive equations for the linear theory for isotropic bodies;
- Finally, we have obtained a set of inequalities for the constant constitutive coefficients of isotropic linear theory that ensure the uniqueness of the solution of the mixed initial-boundary value problem.

The future developments we are working on are the establishment of a reciprocity theorem and a variational formulation of the theory.

## Data Availability

Not applicable.

## References

[1] Cattaneo C. (1948). Sulla conduzione del calore. Atti Sem. Mat. Fis. Univ. Modena.

[2] Cattaneo C. (1958). Sur une forme de l’equation de la chaleur eliminant la paradoxe d’une propagation instantantee. Comptes Rendus.

[3] Vernotte P. (1958). Les paradoxes de la theorie continue de l’equation de la chaleur. Comptes Rendus.

[4] Straughan B. (2011). Heat Waves.

[5] Jou D., Casas-Vázquez J., Lebon G. (1996). Extended Irreversible Thermodynamics.

[6] Müller I., Ruggeri T. (1998). Rational Extended Thermodynamics.

[7] Gurtin M.E., Pipkin A.C. (1968). A general theory of heat conduction with finite wave speeds. Arch. Ration. Mech. Anal..

[8] Chandrasekharaiah D.S. (1998). Hyperbolic thermoelasticity: A review of recent literature. Appl. Mech. Rev..

[9] Chandrasekharaiah D.S. (1986). Some theorems in generalized micropolar thermoelasticity. Arch. Mech..

[10] Green A.E., Laws N. (1972). On the entropy production inequality. Arch. Ration. Mech. Anal..

[11] Ieşan D. (2004). Thermoelastic Models of Continua.

[12] Passarella F., Tibullo V., Zampoli V. (2013). On microstretch thermoviscoelastic composite materials. Eur. J. Mech..

[13] Toupin R.A. (1962). Elastic materials with couple-stresses. Arch. Ration. Mech. Anal..

[14] Toupin R.A. (1964). Theories of elasticity with couple-stress. Arch. Ration. Mech. Anal..

[15] Mindlin R.D. (1964). Micro-structure in linear elasticity. Arch. Ration. Mech. Anal..

[16] Toupin R.A., Gazis D.C. (1965). Surface effects and initial stress in continuum and lattice models of elastic crystals. Lattice Dynamics.

[17] Ahmadi G., Firoozbakhsh K. (1975). First strain gradient theory of thermoelasticity. Int. J. Solids Struct..

[18] Batra R.C. (1976). Thermodynamics of non-simple elastic materials. J. Elasticity.

[19] Kalpakides V.K., Agiasofitou E.K. (2002). On material equations in second gradient electroelasticity. J. Elasticity.

[20] Mindlin R.D., Eshel N.N. (1968). On first strain-gradient theories in linear elasticity. Int. J. Solids Struct..

[21] Ahmadi G. (1977). Thermoelastic stability of first strain gradient solids. Int. J. Non Linear Mech..

[22] Ieşan D. (1983). Thermoelasticity of nonsimple materials. J. Therm. Stress..

[23] Ciarletta M., Ieşan D. (1989). On the nonlinear theory of nonsimple thermoelastic bodies. J. Therm. Stress..

[24] Aouadi M., Ciarletta M., Tibullo V. (2019). Analytical aspects in strain gradient theory for chiral Cosserat thermoelastic materials within three Green-Naghdi models. J. Therm. Stress..

[25] Aouadi M., Passarella F., Tibullo V. (2020). Exponential stability in Mindlin’s Form II gradient thermoelasticity with microtemperatures of type III: Mindlin’s II gradient thermoelastic. Proc. R. Soc. A.

[26] Aouadi M., Amendola A., Tibullo V. (2020). Asymptotic behavior in Form II Mindlin’s strain gradient theory for porous thermoelastic diffusion materials. J. Therm. Stress..

[27] Bartilomo V., Passarella F. (1997). Basic theorems for nonsimple thermoelastic solids. Bul. Inst. Politeh. Iaşi. Secţ. I. Mat. Mec. Teor. Fiz..

[28] Eringen A.C. (2004). Electromagnetic theory of microstretch elasticity and bone modeling. Int. J. Eng. Sci..

[29] Truesdell C., Toupin R., Flügge S. (1960). The classical field theories. Handbuch der Physik.

[30] Parkus H. (1972). Magneto-Thermoelasticity.

[31] Grot R.A., Eringen A.C. (1976). Relativistic continuum physics: Electromagnetic interactions. Continuum Physics.

[32] Nowacki W., Brulin R.H.O. (1983). Mathematical models of phenomenological piezo-electricity. New Problems in Mechanics of Continua.

[33] Maugin G.A. (1988). Continuum Mechanics of Electromagnetic Solids.

[34] Mindlin R.D., Muskilishivili N.I. (1961). On the equations of motion of piezoelectric crystals. Problems of Continuum Mechanics.

[35] Eringen A.C., Maugin G.A. (2012). Electrodynamics of Continua I: Foundations and Solid Media.

[36] Ieşan D. (2018). A theory of thermopiezoelectricity with strain gradient and electric field gradient effects. Eur. J. Mech. A Solids.

[37] Nowacki W. (1978). Some general theorems of thermopiezoelectricity. J. Therm. Stress..

[38] Morro A., Straughan B. (1991). A uniqueness theorem in the dynamical theory of piezoelectricity. Math. Methods Appl. Sci..

[39] Zanchini E., Beretta G.P. (2014). Recent Progress in the Definition of Thermodynamic Entropy. Entropy.

[40] Costanzo L., Lo Schiavo A., Sarracino A., Vitelli M. (2021). Stochastic Thermodynamics of a Piezoelectric Energy Harvester Model. Entropy.

